# Simultaneous High-Frame-Rate Acoustic Plane-Wave and Optical Imaging of Intracranial Cavitation in Polyacrylamide Brain Phantoms during Blunt Force Impact

**DOI:** 10.3390/bioengineering11020132

**Published:** 2024-01-29

**Authors:** Eric J. Galindo, Riley R. Flores, Ricardo Mejia-Alvarez, Adam M. Willis, Michaelann S. Tartis

**Affiliations:** 1 Department of Chemical Engineering, New Mexico Institute of Mining and Technology, Socorro, NM 87801, USA; eric.galindo@student.nmt.edu (E.J.G.); riley.r.flores.th@dartmouth.edu (R.R.F.); 2 Department of Mechanical Engineering, Michigan State University, East Lansing, MI 48824, USA; rimejal@egr.msu.edu (R.M.-A.); adam.matthew.willis@gmail.com (A.M.W.); 3 59th Medical Wing, Office of the Chief Scientist, Lackland AFB, San Antonio, TX 78236, USA

**Keywords:** cavitation, plane-wave imaging, traumatic brain injury (TBI), cranial phantoms, shockwaves, polyacrylamide

## Abstract

Blunt and blast impacts occur in civilian and military personnel, resulting in traumatic brain injuries necessitating a complete understanding of damage mechanisms and protective equipment design. However, the inability to monitor in vivo brain deformation and potential harmful cavitation events during collisions limits the investigation of injury mechanisms. To study the cavitation potential, we developed a full-scale human head phantom with features that allow a direct optical and acoustic observation at high frame rates during blunt impacts. The phantom consists of a transparent polyacrylamide material sealed with fluid in a 3D-printed skull where windows are integrated for data acquisition. The model has similar mechanical properties to brain tissue and includes simplified yet key anatomical features. Optical imaging indicated reproducible cavitation events above a threshold impact energy and localized cavitation to the fluid of the central sulcus, which appeared as high-intensity regions in acoustic images. An acoustic spectral analysis detected cavitation as harmonic and broadband signals that were mapped onto a reconstructed acoustic frame. Small bubbles trapped during phantom fabrication resulted in cavitation artifacts, which remain the largest challenge of the study. Ultimately, acoustic imaging demonstrated the potential to be a stand-alone tool, allowing observations at depth, where optical techniques are limited.

## 1. Introduction

Traumatic brain injuries (TBIs) from blunt and blast-related impacts are prevalent in everyday activities, causing disability and death in civilians and military personnel [1,2,3,4,5]. Over two million TBIs occur in civilian life every year in the United States. In comparison, several hundred thousand military TBIs occur, with a large percentage caused by improvised explosive devices (IED) [6,7,8,9,10]. Although TBIs are well categorized [4,11,12,13,14], damage mechanisms are poorly understood due to the inability to monitor in vivo human brain deformation during collisions [15]. Therefore, researchers have developed surrogate models both experimentally and in silico to study these mechanisms in the human head during a TBI scenario [16,17,18,19,20,21,22,23,24]. Each model has advantages allowing the observation of a particular mechanism at the cost of other limitations. Here, we focus on observing cavitation, optimizing a head model for optical and acoustic data acquisition.

Intracranial cavitation is a suspected mechanism in TBIs, prone to occur in the contrecoup region of the skull during blunt and blast-impact scenarios [16,17]. This phenomenon is the formation, growth, and collapse of bubbles caused by the response to local pressure differentials in fluid, more specifically, the negative pressure resulting in a drop in the local vapor pressure of the medium [15,18]. Cavitation is hypothesized to occur near tissue fluid interfaces such as sulcal, perivascular, and periventricular regions, resulting in high tissue strain rates [18,19,20,21,22,23]. Shockwaves can originate from the violent collapse of cavitation bubbles, creating differences in the local shear strain as they propagate through the cerebrospinal fluid (CSF) into the periventricular, perivascular, and cortical surfaces. in vivo animal studies and postmortem human tissue analysis shows damage at the cellular and tissue level due to compression and shear deformation [16,24,25,26,27]. However, there is no direct evidence in impact scenarios that cavitation occurs in the CSF [15]. The same applies to mild and blunt impacts, where in vivo imaging is not possible in deeper tissue. Therefore, the question remains: Does the CSF cavitate in blunt or blast impacts?

High-speed optical imaging is a powerful method to visualize cavitation bubble dynamics in transparent liquids and soft matter materials [28,29]. One example is the optical inspection of cavitation nuclei, based on the critical radius revealing the effects of local stress and strain, which can assist with brain trauma [30]. This technique allows a high-frame-rate and blur-free observation of transient dynamic events, making it suitable for observing cavitation [31]. Although optical imaging is a popular approach, the lack of penetration depth, where light is scattered, is limiting. Therefore, brain phantoms are restricted by their optical transparency in a high-speed imaging setup, requiring minimal defects so that light can illuminate the imaging plane. Integrating a noninvasive imaging device, such as ultrasound, to observe and quantify cavitation at a depth may assist in understanding cavitation behaviors in various impact scenarios.

Acoustic imaging is well known for detecting stable and inertial cavitation [32,33,34]. Acoustic techniques such as therapeutic ultrasound for drug delivery applications create cavitation where it is detected through a frequency analysis and visualized through passive cavitation imaging [32,35]. A frequency analysis allows the identification of stable cavitation by observing harmonics in reference to a driving frequency. In contrast, inertial cavitation manifests a broadband behavior caused by shockwaves after the cavitation bubble collapse [34,36]. Plane-wave imaging (PWI) is an emerging tool to capture events at high frame rates, up to 10,000 frames per second (FPS), by transmitting and receiving signals with all active transducer elements to produce a single image [37,38,39]. Although PWI can image faster than traditional clinical acoustic methods, the most significant drawback is the lack of transmit focusing, dramatically decreasing spatial resolution with increasing frame rates [37,38,39]. Techniques such as contrast and coherent compound PWI may increase the spatial resolution; however, they either hinder the acquisition frame rate, assume the object of study has no motion, or limit the imaging depth [40,41]. PWI cannot reach the frame rates of high-speed optical imaging; nonetheless, conventional PWI can still be used as a powerful tool to image and potentially detect both stable and inertial cavitation induced by a dynamic load.

This study aims to execute high-frame-rate acoustic PWI to evaluate and address its applicability for intracranial cavitation detection during a blunt TBI scenario using high-speed optical imaging as a validation method. The challenges associated with bubbles trapped during phantom fabrication are discussed as limitations in constructing head models for optical and acoustic cavitation studies. The quantification and observation of cavitation induced by a blunt impact using a drop tower are restricted to a basic head model (lacking a neck replica). This model consists of an extruded two-dimensional 3D-printed skull and a transparent polyacrylamide (PAA) brain tissue model with simplified anatomical features. Early findings suggest acoustic PWI can act as a stand-alone tool for intracranial cavitation detection in the CSF and tissue–fluid interfaces through high-acoustic-contrast regions and bubble behavior presented by a spectral analysis, suggesting its potential for observing cavitation under various impact scenarios.

## 2. Materials and Methods

### 2.1. Formulation of Polyacrylamide Hydrogel Brain Phantoms

PAA compositions of 7% and 10% (*w*/*v*) 60-1 (monomer-to-cross-linker ratio) were chosen to construct a two-layer phantom mimicking the gray and white matter, respectively. The steps for fabricating a brain phantom were similar to those reported previously [42,43,44]. Briefly, degassed DI water was stirred with a monomer (acrylamide (purity ≥ 98.0% (gas chromatography))), cross-linker (N′-Methylenebis (acrylamide) (MBA, purity 99%)), initiator (ammonium persulfate (ACS Reagent, ≥98%)), and catalyst (N,N,N′,N′- tetramethylethylenediamine (TEMED, ReagentPlus, 99%)). The homogenized solution was left to polymerize in a mold, and the phantom was left to swell in an isotonic solution (ISOTON® II Diluent, Beckman Coulter, Radnor, PA, USA) for 24 h at room temperature (21 °C) to simulate the CSF [45]. All chemical substances were purchased from Sigma-Aldrich, Burlington, VT, USA. Both PAA formulations were tested for Shore OO hardness in accordance with ASTM D2240 after the swelling period for each fabricated phantom [46]. These PAA hydrogels were previously characterized to determine swelling, mechanical, and rheological properties for their applicability to simulate brain tissue [42,43,44].

### 2.2. Fabrication and Assembly of the Head Models

**Figure 1:**
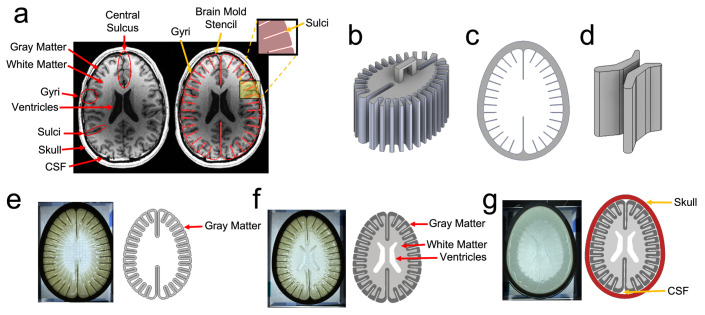

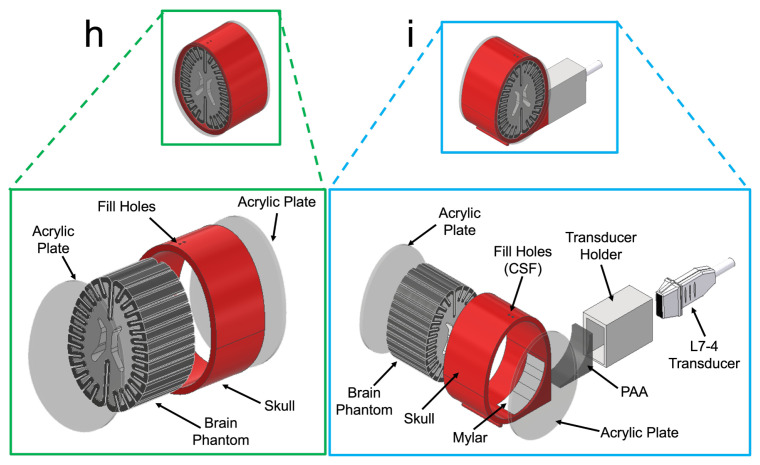
Design, fabrication, and assembly of the human head phantom. The geometrical basis for 3D-printed molds was a 2D transverse planar slice of the human head (**a**) revealing key anatomical features. The 3D-printed mold reveals the (**b**) white matter insert, (**c**) gray matter mold, and (**d**) ventricles. The pouring steps are portrayed by the (**e**) gray matter representation and the (**f**) combination of white and gray matter with a ventricular cavity. The assembly is displayed by the (**g**) PAA phantom sealed in the skull. Lastly, the two different skull geometries reveal the (**h**) original skull model and a (**i**) modified skull model with a transducer port.

Skull and brain molds with simplified anatomical features were designed in Autodesk Inventor based on a transverse planar slice of a magnetic resonance image (MRI) of the human brain displaying brain matter size and geometry (Figure 1a) [29,42,43]. All models were 3D-printed with a Creality CR-10 V3 (Creality 3D Technology Co, Ltd., Shenzhen, China) using a polylactic acid (PLA)-based filament purchased from Hatchbox 3D. The printing parameters are presented in Table 1. Before adding the PAA solution to the brain molds, a releasing agent was applied to minimize possible hydrogel adhesion to the inner walls of the mold. A white matter insert (Figure 1b) was placed inside of a gray matter mold (Figure 1c) where a 7% (*w*/*v*) 60-1 PAA formulation was poured, representing the gray matter layer (Figure 1e). The insert was then removed where 3D-printed ventricles (Figure 1d) were centered inside the gray matter mold in which a 10% (*w*/*v*) 60-1 PAA composition was then poured as the white matter layer (Figure 1f). The fabricated two-layer phantom was allowed to swell in an isotonic solution. Once the swelling period was over, it was sealed inside one of the skull geometries (Figure 1g–i) with an isotonic solution where 1/4″ thick acrylic plates were adhered with a thermoplastic adhesive. This procedure was similar to a single-layered phantom, except a 10% (*w*/*v*) 60-1 PAA composition was used without the white matter insert. Lastly, rubberized tape sealed the fill ports on top of the skull.

### 2.3. Drop Tower Assembly for Blunt Impacts

**Figure 2:**
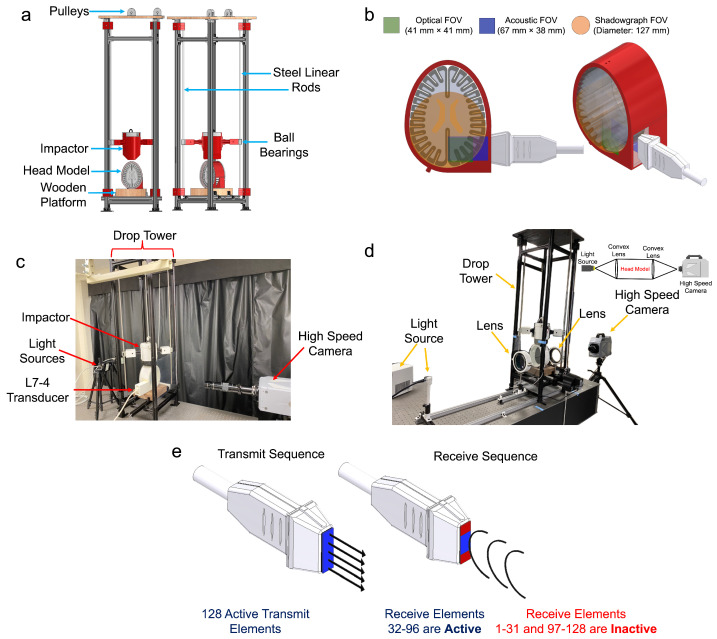
Schematic of the (**a**) drop tower assembly, (**b**) different fields of view for each imaging setup, (**c**) optical imaging in conjunction with acoustic imaging, (**d**) shadowgraph imaging, and (**e**) transmit and receive acoustic data acquisition sequence.

A drop tower (Figure 2a) was designed in Autodesk Inventor and fabricated to mimic blunt impacts inducing cavitation inside head models similar to Kerwin et al. [29]. The drop tower was comprised of an aluminum T-slot railing held together by T-slot L-brackets. Steel linear rods enabled the impactor to drop from specified heights using low-friction, high-strength ball bearings. A Longer LK1 (Longer 3D Technology, Shenzhen, China) 3D printer with PLA filament (Hatchbox 3D, Rowland Heights, CA, USA) printed the impactor with a hollow internal structure and planar bottom edge with the printing parameters presented in Table 1, allowing it to be filled with a user-defined mass, where a lid then enclosed the printed structure. The impactor was raised by a rope via a pulley system and mechanically released by an actuator, breaking a 36 AWG nichrome wire and triggering the imaging hardware. This impacted the head model, which was positioned on a rigid walnut wood block centered and held in place by T-slot L-brackets. The downward linear velocity of the descending impactor was approximated through high-speed optical imaging, with the bottom of the impactor set as the point of reference. The impact energy was determined as the kinetic energy (KE) at the point of impact through Equation (1), where *m* is the mass and *v* is the velocity. The peak impact force (*F*) was determined by Equation (2) where the maximum skull displacement (*d*) was measured at the top surface of the skull until it rebounded, determined through high-speed optical imaging and Matlab image pixel tracking.

(1) $$KE=\frac{1}{2}mv^{2}$$

(2) $$F=\frac{2KE}{d}$$

### 2.4. High-Speed Optical Imaging of Cavitation in Two Skull Geometries, with and without a Transducer Port

A similar setup to Figure 2c allowed for a full field of view (FOV) of the skull to be observed under impact with a Photron SA-Z high-speed camera recording at 20,000 FPS and a shutter speed of 48.4 μs, where each frame attained a 1024 × 1024 image resolution. A Nikon AF-S DX Zoom Nikkor 18–55 mm (Nikon, Tokyo, Japan) zoom lens and a GS Vitec MultiLED G8 (GS Vitec, Bad Soden-Salmünster, Germany) light source with two MultiLed QT LEDs attached with a 60° wide angle lens above 80% power provided sufficient lighting to enable imaging. Both skull geometries were filled with DI water or isotonic solution with a brain phantom. The total cavitation bubble area was monitored in Matlab using the image processing toolbox on a frame-by-frame basis. Image processing was performed by utilizing a two-dimensional fast Fourier transform (FFT) applied onto grayscale frames with a high-pass Gaussian filter covering the highest frequency of the centered FFT spectra for cavitation bubble segmentation, which was then converted to a binary image for a pixel area calculation. This task was performed as a two-part process where initial and secondary processes segmented the cavitation growth within the medium and the inside curvature of the skull, which were then added to create a final frame.

### 2.5. Shadowgraph Imaging for Cavitation and Shockwave Visualization

Shadowgraph imaging was performed under a parallel light field constructed by two Wollensak MTD telescope objective lenses with diameters of 127 mm and a focal length of 700 mm, where the FOV of the lenses is presented in Figure 2b. A SugarCUBE Ultra LED Illuminator light source illuminated the parallel field. The drop tower and head model were placed between both lenses within the parallel field (Figure 2d). A Photron SA-Z high-speed camera recording at 100,000 FPS with a 0.16 μs shutter speed (640 × 280 image resolution) captured events within the parallel light field.

### 2.6. High-Frame-Rate Acoustic Plane-Wave Imaging in Conjunction with Optical and Shadowgraph Imaging

The optical imaging setup is presented in Figure 2c where a DistaMax K-92635 (Infinity Photo-Optical, Centennial, CO, USA) lens was used and shadowgraph imaging was kept the same as Figure 2d. A Verasonics Vantage 64 ultrasound research scanner (Verasonics, Inc., Kirkland, WA, USA) was paired with a Phillips ATL L7-4 linear array transducer involving a PWI programming script with an acquisition frame rate of 8620 FPS, a transmit voltage of +17.0 V, and a transmit frequency of 5.2 MHz. The reconstruction of all acquisitions to pixel data involved using Verasonics built-in Matlab processing structure with minimal gain and excluding contrast enhancers. All reconstruction parameters were kept constant across all frames, so the contrast shown from a reconstructed frame was at a constant pixel intensity. A high-quality wide-beam and b-mode ultrasound image of the sealed brain phantom was taken to identify any pre-existing artifacts before and after the impact. Both ultrasound techniques utilized an axial and lateral depth of 67 mm and 38 mm (Figure 2b) to attain 128 transmit and 64 receive elements (Figure 2e), respectively. The Verasonics hardware and the high-speed optical camera were triggered simultaneously, similar to Section 2.3, to compare optical and acoustic frames using a common relative time frame.

### 2.7. Acoustic Spectral Analysis and Cavitation Mapping

**Figure 3:**
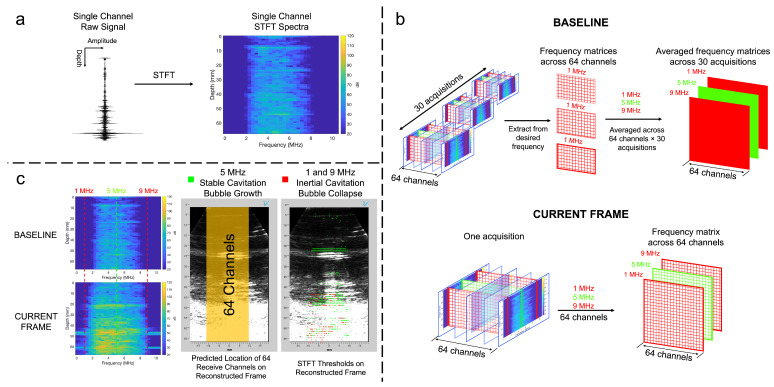
Cavitation mapping entailed the (**a**) creation of a spectral map by transforming the raw signal of a signal channel through Matlab’s built-in STFT function, (**b**) the fabrication of a baseline and comparison to a frame of interest by extracting data based on selected user-defined frequencies, and (**c**) a comparison of baseline and a selected acquisition for mapping potential regions of stable and inertial cavitation onto a reconstructed plane wave frame.

An acoustic spectral analysis was performed on the averaged raw signal across all 64 channels and a single user-defined channel through a power linear and logarithmic approach. Cavitation mapping involved transforming the raw time-domain signal of an individual channel into Matlab’s built-in short-time Fourier transform (STFT) function (Figure 3a) with inputs shown in Table 2 mapped with an amplitude logarithmic scale. To measure differences between STFT spectral maps, a baseline composed of 30 acquisitions before impact was created by extracting data from each desired frequency (1, 5, and 9 MHz) for all 64 channels in a single acquisition, in which 30 acquisitions were averaged to obtain a baseline matrix for 1, 5, and 9 MHz (Figure 3b). The baseline was then compared to singular acquisitions of interest with the same desired frequencies where a threshold of 30 dB increase at 5 MHz and 10 dB increase at both 1 and 9 MHz indicated stable and inertial cavitation, respectively (Figure 3c). Lastly, the approximated location of the 64 central transducer elements allowed for a mapping of potential stable (green square) and inertial cavitation (red square).

## 3. Results

### 3.1. Drop Tower Characterization Based on Mass and Drop Height

Table 3 shows the drop tower impact parameters as a function of varying drop heights and impactor mass. The impactor was not treated as a free-falling object, but as a low friction with linear displacement. As expected, all parameters increased with the drop height and mass. The impact energy, skull displacement, and peak impact force obtained a higher variance with the increasing mass. All parameters were tested on head models incorporating an original skull geometry to determine if cavitation was generated. The impact parameters that generated the greatest amount of cavitation were applied to a head model incorporating the modified skull which accommodated an ultrasound transducer.

### 3.2. Validation and Comparison of Cavitation Behavior with a Change of Skull Geometry to Accommodate an Ultrasound Transducer

In order to determine a critical drop mass and height to reproducibly achieve cavitation in each skull geometry, the bubble area was calculated with Matlab’s image processing toolbox, as presented in Figure S1. In general, impact energies below 4.2 J produced a bubble area below 9 mm^2^, where minor bubbles gave rise to a single peak at 650–1550 μs. Impact energies above 4.5 J produced a total bubble area with two emerging peaks displayed at 1000–2000 μs and 1700–2300 μs, with the 4 kg, 60 cm impact parameters providing the greatest total bubble area. By focusing on 4 kg and 60 cm, the first peak was attributed to a first round of cavitation when observing optical frames of the same test (Figure S1b), where bubbles grew towards the bottom region of the skull, as shown by the yellow arrows (t = 1400 μs). The second peak corresponded to the creation of a second round of cavitating bubbles (t = 2500 μs), while the first round was still during its bubble collapse phase. Since an impactor mass of 4 kg and a drop height of 60 cm provided the highest total bubble area, these parameters were used in all other experiments. The cavitation pattern for a two-layered phantom was similar to DI water but with less bubble content, with bubbles near the gel interface, the acrylic plate, and 3D-printed skull, as presented in Figure S1d. A second geometry with a port to accommodate the L7-4 linear array transducer was tested to ensure cavitation activity.

**Figure 4:**
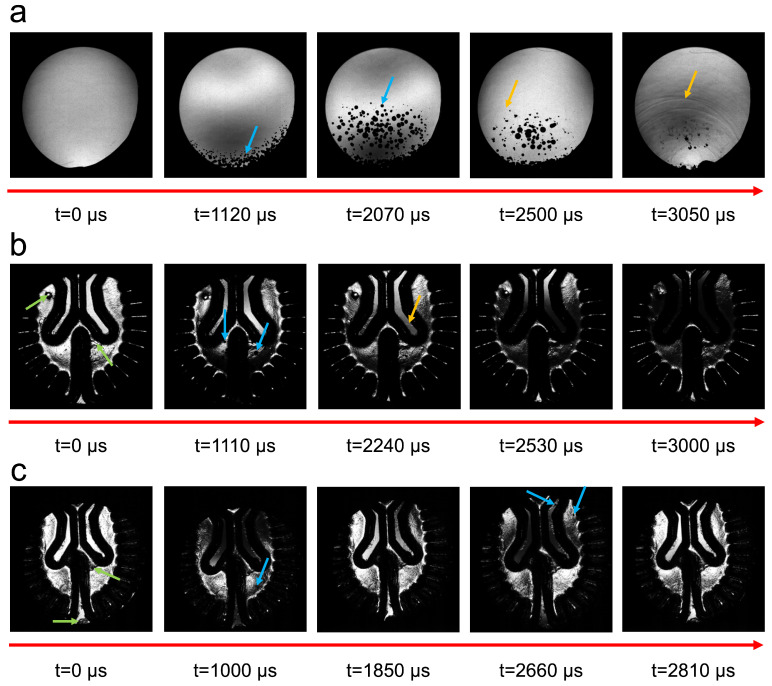
Shadowgraph imaging of head models illustrating cavitation bubble growth and collapse in the original skull geometry without an ultrasound port filled with (**a**) DI water (Video S2), (**b**) single-layered phantom (Video S3), and (**c**) a two-layered phantom (Video S4) all impacted with an impactor mass of 4 kg and a drop height of 60 cm. The green arrows display pre-existing bubbles before impact, the blue arrows represent areas of bubble growth, and the orange arrows reveal shockwave locations.

Shadowgraph imaging provided cavitation bubble dynamics and shockwave visualization. Figure 4 showcases one representative image from each experiment. The DI water medium illustrated the best representation of the cavitation phenomenon due to its transparency and adequate sealing method. An inadequate sealing method can be visualized in Video S1, displayed by many pre-existing bubbles before impact. Figure 4a displays the first round of cavitation in the DI water medium at 1120 μs after impact with bubble growth near the bottom region of the skull as presented by the blue arrow. Similarly, at 2070 μs, a second round of cavitation bubbles appeared, as shown in Video S2. As time progressed, bubbles collapsed, and shockwaves appeared, indicated by the orange arrows displayed on the 2500 μs and 3050 μs frames.

Surface aberrations from the polymerization process are shown by the green arrows in Figure 4. The tissue–fluid interfaces of the phantoms were visualized as dark regions within the hydrogel near the sulcal regions and ventricles, hindering the observation of cavitation. However, cavitation was still visible at 1110 μs, as indicated by blue arrows. Shockwaves were challenging to view during bubble collapse with the polyacrylamide brain. However, they were propagating from the contrecoup to the coup region of the head model in relation to the impact as shown in the ventricular cavity at t = 2240 μs (Video S3). From 2530 to 3000 μs, dark regions across the entire frames were driven by elastic deformations in the skull and acrylic plates. The shadowgraph frames in a two-layer phantom presented in Figure 4c had less optical transparency than a single layer, where surface imperfections and defects in the hydrogel were present. Bubble growth was visualized near the same time. Interestingly, at 2660 μs, cavitation in the form of a small dark bubble was seen inside and near the ventricular cavity. Shockwaves were less visible but still occurred with a two-layered phantom, also propagating from the contrecoup to the coup region (Video S4).

### 3.3. Comparison of Acoustic Plane-Wave Imaging and Optical Imaging for Intracranial Cavitation Detection with Varying Numbers of Pre-Existing Bubbles

**Figure 5:**
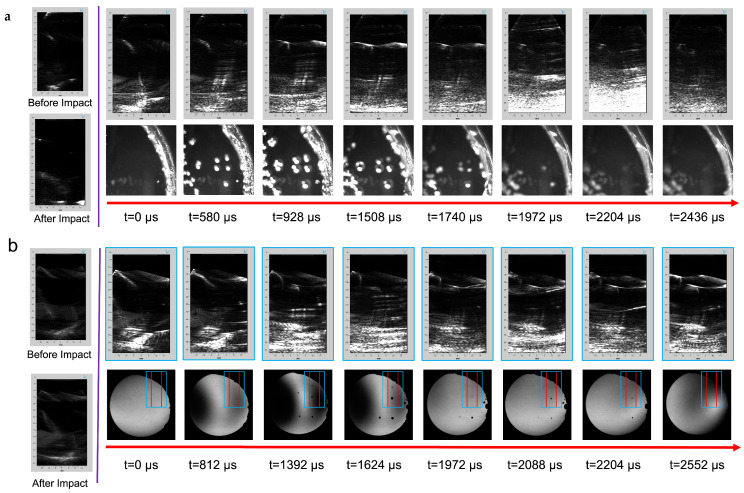
Acoustic and both optical and shadowgraph imaging timelines of head models incorporating a modified geometry to accommodate an ultrasound transducer filled with DI water impacted with an impactor mass of 4 kg and a drop height of 60 cm where one model (**a**) has more pre-existing bubbles (Video S5) compared to (**b**) a better sealing method (Video S6). The blue box corresponds to the 128-element transmit plane wave, while the area between the red lines correlates with the 64 receive elements.

High-quality ultrasound imaging enabled the visualization of pre-existing bubbles before each impact test. Pre-existing bubbles were present in each drop tower test obtained through wide beam (Figure 5a) or b-mode (Figure 5b) imaging. The comparison of high-quality frames before and after impact illustrated that acoustic scatterers increased in contrast at the same location or were removed upon impact. The high-quality frames could be compared to the first plane-wave reconstructed frame (t = 0 μs) where the pre-existing contrast was in the same axial and lateral directions as the high-quality imaging but with more intensity due to the plane-wave reconstruction. In some cases, it was possible to remove bubbles before the impact.

**Figure 6:**
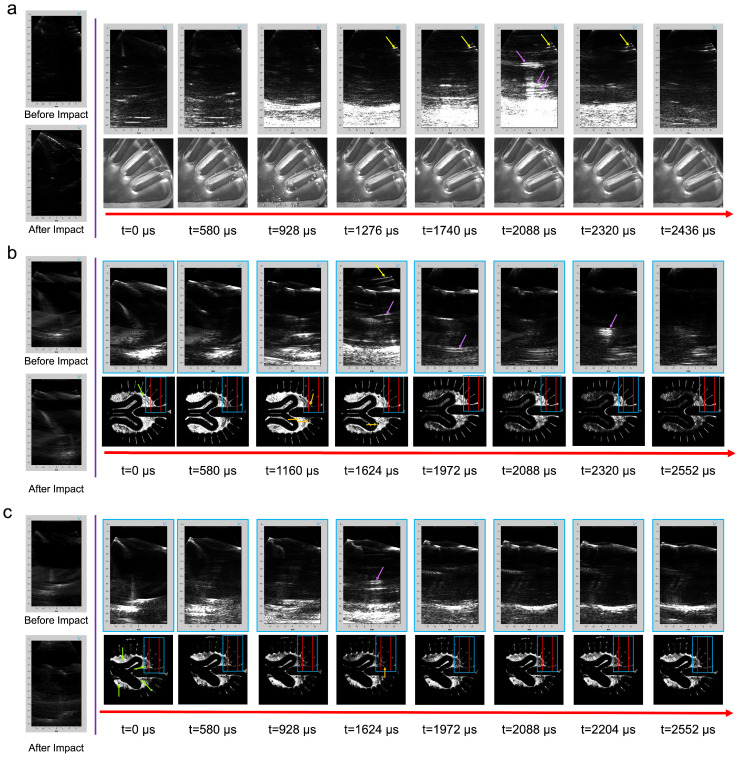
Acoustic and optical imaging timelines of head models incorporating a modified geometry filled with an isotonic solution and a (**a**) two-layer PAA brain with an inadequate sealing method (Videos S7 and S8), (**b**) single-layer phantom, and (**c**) a two-layer phantom impacted with an impactor mass of 4 kg and a drop height of 60 cm. The blue box corresponds to the 128-element plane-wave transmission while the area between the red lines correlates with the 64 receive elements. Green arrows display areas of pre-existing defects in the PAA gel, while orange arrows represent areas of bubble growth. Yellow arrows display the reverberation in the PAA standoff pad, whereas magenta arrows highlight the reverberation generated from multiple bubbles within the head model.

A general trend of events is presented for head models filled with and without a brain phantom (Figure 5 and Figure 6). For all tests, cavitation bubbles began to grow and reach their maximum size at 580–1508 μs. During that period, PWI revealed an increase in intensity in the far field where the central sulcus was located as a prominent intensity in the midfield of the transducer. Bubbles then collapsed, and shockwaves propagated throughout the head model at 1740–3000 μs. From an acoustic perspective, bubble collapse was visualized as an increase in intensity in the reconstructed frame. Figure 5a presents bubble growth, where bubbles were spaced closely (580–1508 μs) in Video S5. During the same time frame, the plane-wave frames showed a reverberation between bubble sources before they disappeared upon bubble collapse.

A better sealing method was developed to address the abundance of trapped or pre-existing bubble artifacts. Video S6 shows decreased cavitation from bubbles trapped in the fabrication process, enabling a clearer visualization of cavitation from impact (Figure 5). By observing t = 1392 μs, two bubbles were captured within the acoustic FOV with a defined contrast near the lateral midplane with a similar reverberation artifact present at t = 1624 μs. Lastly, an emission of a shockwave from a bubble collapse is shown in both optical and acoustic frames, where a circular pattern was visualized through PWI at t = 1972 μs near an axial distance of 50 mm.

Head models filled with brain phantoms (Figure 6) presented similar acoustic artifacts to those in a DI water medium, where more pre-existing bubbles altered the quantity of cavitation. These pre-existing bubbles were present near the central sulcus for all phantoms. However, specifically for a two-layer phantom, bubbles were trapped within the PAA gray and white matter interface as presented through optical and acoustic frames. Figure 6a presents a two-layer phantom with pre-existing bubbles with the cavitation growth and collapse phase that occurred more quickly than those with minimized pre-existing bubbles. Therefore, a large amount of acoustic contrast was present in the far field (t = 928–1740 μs). During bubble collapse (t = 1392 μs), a spike of intensity ranged across the axial dimension of the acoustic FOV. The one- and two-layer phantoms both had increased intensity albeit reduced compared to models without a brain phantom (Figure 6b) in the axial direction of the midfield at t = 1624 μs; however, the contrast was drastically reduced.

### 3.4. Acoustic Spectral Analysis and Cavitation Mapping

**Figure 7:**
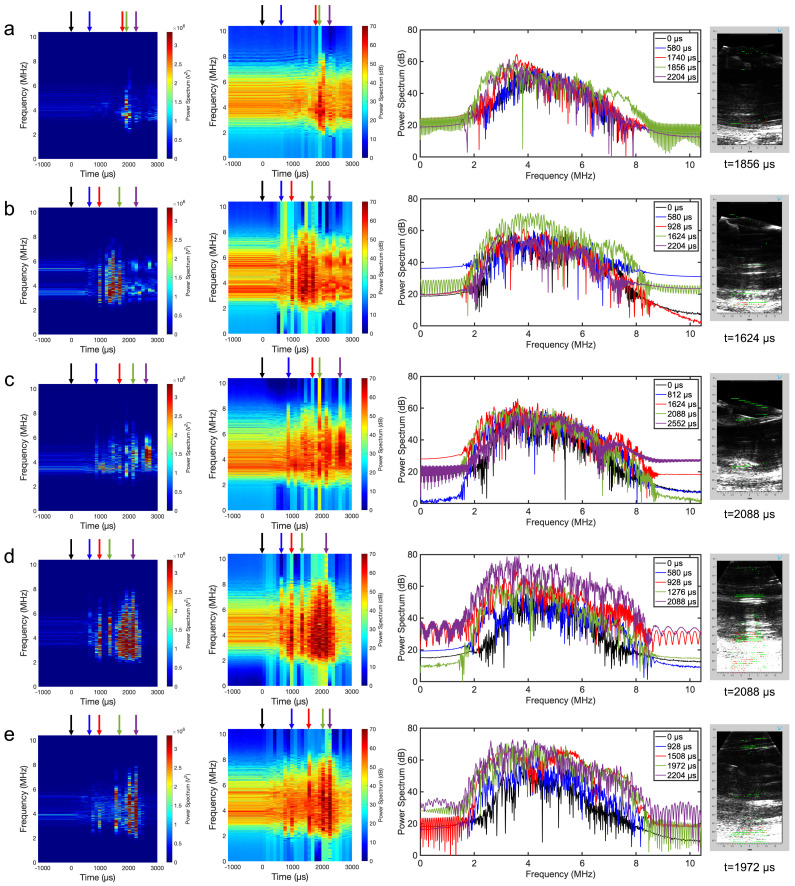
Acoustic spectral analysis regarding a user-defined single channel linear and log power spectrograms, time-dependent logarithmic spectra, and cavitation mapping on a reconstructed frame. The following test involves a better sealing method and goes as follows: (**a**) channel 34 of a one-layer brain phantom, (**b**) channel 11 of a two-layered phantom, and (**c**) DI water. Both tests regarding (**d**) channel 47 of a two-layered phantom and (**e**) channel 24 of a DI water correspond to tests with a higher number of pre-existing bubbles.

The acoustic spectral analysis enabled a different approach to confirming the presence of cavitation as opposed to relying on qualitative observations of both optical and plane-wave frames presented in Figure 5 and Figure 6. Initially, the spectral analysis was performed on the average of all 64 receive channels as a simple way to capture cavitation behavior over time (Figure S3). Although an effective approach, the bubble response was not well represented since the averaged signal muted key features of the spectra. Therefore, receive signals from a select channel were used to detect cavitation, as presented in Figure 7. The single-channel linear power spectrograms represent the overall acoustic energy of the impact. For all tests, minor variances occurred before impact (t < 0 μs), indicating the presence of pre-existing bubbles trapped in the head model. At t = 0–1500 μs, there was an increase in power caused by bubble growth, but shockwaves from bubble collapse (t > 1500 μs) produced the highest amount of energy in the spectrograms. The logarithmic power spectra provided a similar result across all tests by defining the existence of a broadband behavior across all frequencies but allowed the visualization of broadband components. During bubble growth, the spectrum did not differ in behavior from its baseline (t = 0 μs). However, its energy increased when cavitation bubbles reached their maximum size before transitioning to a collapse phase. During bubble collapse, referring to bubble clouds and shockwaves, the spectrum was elevated in energy across all frequencies compared to its baseline shown at t = 1856 μs and t = 2088 μs in Figure 7c and d, respectively. The STFT map was overlaid onto reconstructed frames in an attempt to localize areas of cavitation. For all head models, stable cavitation was displayed in the near field (green pixels), with inertial cavitation displayed dominantly in the far field of the reconstructed frame with a high acoustic contrast. For head models with a reduction in pre-existing bubbles, the single-layer phantom (Figure 7a) showed less cavitation than head models incorporating either a two-layer phantom or DI water alone (Figure 7b,c). This is likely due to the two-layered phantom pouring process that trapped small bubbles between each layer (Figure 7d) resulting in stable cavitation being mapped throughout the image. Similarly, the DI water (Figure 7e) medium showed more cavitation with more contrast presented in the far field compared with the optimized phantom with fewer pre-existing bubbles.

## 4. Discussion

### 4.1. Benefits and Limitations of PAA for Mimicking Brain Tissue

PAA was chosen to model brain tissue based on practical features, such as tunable mechanical properties, room temperature casting, curing ability, and optical transparency. PAA is a polymeric hydrogel chemically constructed through a free-radical polymerization reaction where an imperfect network between water and cross-linked acrylamide chains is formed and is known to mimic biological tissues. It is also suitable for most optical and acoustic imaging techniques [33,47]. The PAA phantoms mimicked the human brain’s gray and white matter tissue by attaining a similar shear modulus under low and high dynamic shear rates conducted by classical oscillatory parallel plate rheometry and magnetic resonance elastography [43,44,48]. The experimental density measurements of the swollen gray and white matter components were 1005 ± 85 kg/m^3^ and 1005 ± 47 kg/m^3^, respectively, making it comparable to human (≈1081 kg/m^3^) and porcine brain matter (≈1040 kg/m^3^) [49,50,51]. For quality control and consistency between multiple phantom fabrications, a Shore OO durometer determined a hardness value intended for soft biological tissues [46]. Therefore, the 10% (*w*/*v*) 60-1 PAA formulation provided a hardness value between 10.4 and 13.2 after a 24 h swelling period in an isotonic solution (Table S1), which is close to animal brain tissue or other brain tissue simulants with comparable shear rheological properties [52]. The 7% (*w*/*v*) 60-1 PAA formulation was too soft and did not produce enough mechanical resistance to establish a Shore OO hardness value.

PAA has limitations, which affected cavitation and the anatomical features of the brain phantom. The first complication was that bubbles could be trapped between and within the gray and white matter layers during the fabrication of the two-layer phantom. Bubbles in the isotonic media could also be trapped between the sulcal regions, resulting in acoustic artifacts. An extreme case is displayed in Figure S4. To minimize the issue of pre-existing bubbles, a single-layer phantom was explored, reducing the possibility of trapped bubbles during the polymerization process. The second issue was that the 60-1 PAA hydrogels swelled rapidly. Therefore, the swelling period was 24 h postproduction since most of the volumetric growth occurred within one day after exposure to water or isotonic solution [44]. However, conducting impact experiments within a short time period is vital for maintaining sulcal widths that match the in vivo human brain while maintaining a gap between the phantom, 3D-printed skull, and acrylic plates for freedom of movement and rotation. The approximated sulcal widths of the brain phantoms was uniformly 1 mm, which is close to middle-aged adults (1.27 ± 0.17 mm) and the range of sulcal widths across different regions of the brain (1.0–2.5 mm) [53]. The gap between the phantom and the acrylic plates and the phantom and the skull was roughly 1–3 mm. Therefore, controlling the phantom’s volumetric swelling is necessary since it will increase in volume, eliminating a slip boundary, warping the ventricles, enlarging the gyri, altering the sulcal widths, and modifying the shear properties of the PAA hydrogel [54,55]. Due to this swelling restriction, a new brain phantom must be fabricated after a 24 h swelling period.

### 4.2. Head Model Characteristics

To enable acoustic and optical image acquisition of cavitation, we modified a previously developed head phantom and drop tower setup [29]. Figure 1i displays a similar geometry but modified by adding a rectangular right-angle extrusion to insert a Phillips ATL L7-4 linear array ultrasound transducer. The modified skull geometry required a 10% (*w*/*v*) 60-1 PAA gel to fill the remaining space and couple the transducer for optimal image quality by reducing acoustic scattering from the coupling gel. Ultimately, despite the change in geometry, cavitation was still observed.

The skull models were limited to easily accessible and low-cost materials for continuous repeatable use of the 3D-printed parts. The head model was mainly composed of PLA manufactured by Hatchbox with mechanical and material properties comparable to postmortem human cranial bone in the form of density and elastic modulus in a room temperature environment [56,57,58]. The skulls were printed with the parameters listed in Table 1 chosen based on the tensile and flexural strength for a specimen printed in the flat orientation [59,60]. PAA has a lower flexural strength than the human skull and endures more strain with load [44]. Since 3D-printed skulls fail at higher strains with no visible signs of plastic deformation, they were reused for cavitation studies regarding the impact parameters presented in Table 3.

Several parameters relevant to human cranial anatomy were mimicked. The thickness of the 3D-printed skull was nearly 8 mm, which is close to the average thickness of an elderly postmortem human cranial skull [58,61]. The inner volume of the skull was 1673 (cm^3^), which is higher than the average male and female mesocephalic cranial capacity [62]. Also, the fabricated brain phantom had a mass close to 1380 g, near that of the average adult male [63]. Lastly, the approximate volume of the swollen brain phantom was calculated as 1214 (cm^3^), which falls into the category of an average young, healthy male or female adult [64].

### 4.3. Confirmation of Inducing a Blunt Impact Based on Drop Tower Characterization

The scope of this work was to develop an acoustic tool to detect cavitation in a blunt-impact scenario. To achieve this, we implemented a drop tower to produce mechanical energies that satisfied blunt-impact criteria and reliably produced cavitation without fracturing the skull material [11]. Specifically, impactor velocities were lower than most experimental blunt impacts; however, impact energies surpassed the mechanical energies necessary to fracture a human skull with similar displacement and impact forces, seen in blunt ballistic scenarios with head models or human cadaver skulls [65,66]. The contact time of the impact was 2.2–3.0 ms before the skull rebounded vertically with a reduced contact force, making it consistent with computational modeling of blunt impacts of foreign objects colliding with the human head [67]. A limitation is that the planar impact did not induce a rotational motion of the skull due to a lack of a neck model. It is important to acknowledge that each human TBI is unique, and this scenario models only this particular impact but provides a baseline tool to iterate and apply the model to other scenarios in future work.

### 4.4. Minimizing Trapped Bubbles during the Assembly Process

Minimizing artifacts from pre-existing bubbles is essential to avoid image processing errors. While baseline subtraction is possible, the bubbles become sound sources, making it difficult to separate them from cavitation bubbles during the impact event. Examples of these artifacts are shown in Figure S5. The head models were filled by a batch process, allowing surface air bubbles to cling to the surface of the acrylic plate via surface tension. Manual agitation was applied to remove the bubbles from the acrylic plate, which integrated some of those air bubbles into the fluid, creating an artificial cavitation event in the contrecoup region of the head model. Finally, there were minor surface imperfections in the manufacturing process of cast acrylic, which enabled air bubbles to adhere to the surfaces of the plate (Video S9) [68]. A better sealing method was introduced to minimize the appearance of these artifacts. This method eliminated the batch-filling process by slowly integrating a continuous pour of solution into the head model before it was sealed with an acrylic plate and topped off through the fill ports. Before integrating the CSF simulant, the acrylic plate and printed skull were sprayed with 70% isopropyl alcohol to minimize the surface tension. Lastly, a continuous bead of adhesive allowed for a better seal versus a discontinuous bead around the perimeter and interface of the acrylic sheet and skull.

### 4.5. Cavitation Comparison of Transducer Port Skull Modification with High-Speed Optical Imaging

High-speed optical imaging served as a validation tool for cavitation, providing the time frame of bubble growth and collapse with respect to the impact. This allowed comparisons of intensity regions imaged by PWI. Image processing provided a simplistic and rapid approach to determining the total bubble area but had two main limitations. The first is that the Matlab script excluded any black pixels within the bubble from the pixel area calculation, occurring from bubble transparency where no artificial binary fillers were implemented. The second drawback is that cavitation bubbles had the same light intensity as the hydrogel phantoms, making segmenting bubbles through traditional image processing functions challenging. The main limitations of traditional high-speed imaging are a lack of shockwave visibility and a fixed single imaging plane. However, shadowgraph imaging provided a qualitative approach with more depth of information, and shockwave dynamics of different mediums, based on the significant differences in refractive index [69]. The trade-off was that the imaging plane collapsed into a two-dimensional image. Therefore, the spatial depth of a bubble could not be determined. Shadowgraph imaging performed best with head models filled with DI water, where the effects of the impact were well observed. The shockwave velocity resulting from a violent collapse of a bubble was not determined. However, this phenomenon is well studied where the primary and secondary shock velocity originating from a single- or double-cavitation bubble collapse can grow outwards at a shockwave speed of 1590 m/s before it starts reducing in velocity [70,71]. Cavitation bubbles and shockwaves were visible in the biofidelic PAA phantoms. However, dark regions throughout the phantom caused by surface aberrations made it difficult to visualize. To minimize these defects, it is suggested that phantoms polymerize on smooth surfaces on all sides. Dark regions were present near the tissue–fluid interfaces; polymeric bonds were likely concentrated near the boundaries of the 3D-printed brain molds, which altered the refractive index of the hydrogel and obstructed the view for potential cavitation events. Ultimately, shockwaves were detected in each scenario.

Examining different mediums and geometries through high-speed optical imaging suggests the modified skull geometry to accommodate a transducer did not eliminate cavitation. However, it did affect the timing, appearance, and behavior of cavitation and its respective shockwaves compared to the original skull geometry. In general, when a one- or two-layer phantom is sealed in the head models, less cavitation is present compared to a DI water medium due to the attenuating effects of the PAA hydrogel. This attenuation effect is appropriate for brain phantoms since human brain tissue is known to have a prominent damping ratio [72]. For these studies, intracranial cavitation was focused on the soft matter tissue and the central sulcus regions of the head models. Although optical imaging served as a method for comparing two skull geometries, the modified skull model enabled PWI to study cavitation events through reconstructed frames and the acoustic spectral analysis.

### 4.6. Evaluation of Acoustic Plane-Wave Images for Intracranial Cavitation Detection

Acoustic PWI was implemented as a novel approach to detect intracranial cavitation by providing information at depth compared with high-speed optical imaging. Interestingly, most acoustic cavitation applications are intended for clinical therapeutic ultrasound, where pulses drive bubbles at a targeted location [35,73]. Monitoring and controlling therapeutic cavitation activity is performed with passive cavitation imaging. This approach captures real-time bubble emissions where data reconstruction techniques such as beamforming perform a delay-and-sum algorithm, typical for linear or phased array transducers, to quantify cavitation [32,74,75]. However, in these applications, the location of cavitation is known. In our application, the spatial location and quantity of cavitation are unknown and driven by the impact. Here, the PWI sequence was comprised of two Matlab scripts provided by Verasonics, which acquired and reconstructed separately to preserve the frame rate of 8620 FPS (116 μs/acquisition) and allowed access to all received data for further offline processing.

The acoustic spectral analysis established evidence for the detection of intracranial cavitation. Both logarithmic and linear power spectrograms provided evidence of cavitation with energy jumps during bubble growth and collapse. The logarithmic power spectra at various time points revealed areas of potential harmonic behavior indicating stable cavitation, whereas broadband signals at later time points indicated inertial cavitation from shockwaves. Cavitation mapping, using an STFT, was able to display the location of possible stable and inertial cavitation sites primarily focused in the far field of the transducer. However, the mapping was limited to the approximate location of the 64 receive channels. Acoustic PWI has the potential to serve as a stand-alone tool for intracranial detection but needs improved methods where stable and inertial cavitation can be defined more accurately in terms of spatial location. Several approaches are suggested for combating this issue. The first is improving the resolution of the STFT and defining better energy thresholds at 1, 5, and 9 MHz, which can lead to a more accurate spatial location at the cost of computational time. Also, implementing postreconstruction techniques such as the ones presented in passive cavitation imaging can illustrate the potential location of cavitation based on time-of-flight calculations and energy differences on a frame-by-frame basis [32].

## Figures and Tables

**Table 1:** Head model 3D-printing parameters.

Parameter	Requirement
Infill percentage	100
Infill pattern	Grid
Layer height	0.2 mm
Wall thickness	0.8 mm
Wall line count	2
Top and bottom layers	2
Extruder temperature	205 °C
Build plate temperature	60 °C
Print speed	40 mm/s

**Table 2:** Matlab STFT inputs for the acoustic spectral analysis.

Parameter	Requirement
Spectral window size	64 samples
Spectral window type	Hanning (Periodic)
Overlap length	32 samples
FFT length	64 samples
Frequency range	One-sided
Input vector size	2048 × 1
Output matrix size	63 × 33

**Table 3:** Drop tower impact parameters based on various impactor masses and drop heights at the point of impact (N = 3).

Impactor Mass (kg)	Drop Height (cm)	Velocity (m/s)	Impact Energy (J)	Skull Displacement (mm)	Peak Impact Force (kN)
2	20 40 60	1.28 ± 0.03 1.42 ± 0.06 1.61 ± 0.03	1.65 ± 0.08 2.02 ± 0.18 2.60 ± 0.09	1.26 ± 0.08 1.40 ± 0.12 1.62 ± 0.12	2.60 ± 0.25 2.90 ± 0.05 3.15 ± 0.25
3	20 40 60	1.31 ± 0.05 1.58 ± 0.06 1.74 ± 0.05	2.57 ± 0.20 3.73 ± 0.28 4.54 ± 0.29	1.69 ± 0.07 2.29 ± 0.10 2.60 ± 0.07	3.11 ± 0.37 3.25 ± 0.15 3.43 ± 0.03
4	20 40 60	1.44 ± 0.06 1.71 ± 0.02 1.83 ± 0.09	4.12 ± 0.33 5.82 ± 0.16 6.71 ± 0.63	1.98 ± 0.17 2.83 ± 0.11 2.93 ± 0.27	3.95 ± 0.35 4.11 ± 0.12 4.98 ± 0.74

## Data Availability

The raw data supporting the conclusions of this article will be made available by the authors on request.

## Supplementary Materials

The following supporting information can be downloaded at https://zenodo.org/records/10574244, Figure S1: Comparison of two skull geometries with various impact parameters for cavitation thresholds; Figure S2: Verasonics acoustic plane-wave simulation of 128 transmit and 64 receive channels with acoustic scatterers; Figure S3: Acoustic spectral analysis regarding averaged 64 channels revealing linear and log power spectrograms and time-dependent logarithmic spectra; Figure S4: High-quality wide-beam image of a single- and double-layer PAA brain phantom with pre-existing bubbles; Figure S5; Comparison of sealing methods and their effect on the segmentation of cavitation bubbles through Matlab image processing; Table S1: Shore OO hardness values for 10% (*w*/*v*) 60-1 PAA brain phantoms; Videos S1–S7, S9: High-speed optical imaging of head models filled with DI water, single-layer, or double-layer PAA phantoms; Video S8: Acoustic plane-wave imaging of a 2-layer phantom. Reference [76] are cited in the supplementary materials.

## Abbreviations

The following abbreviations are used in this manuscript:

TBI	Traumatic brain injury
DI	Deionized water
CSF	Cerebrospinal fluid
PWI	Plane-wave imaging
FPS	Frames per second
PAA	Polyacrylamide
MRI	Magnetic resonance imaging
PLA	Polylactic acid
KE	Kinetic energy
FOV	Field of view
FFT	Fast Fourier transform
STFT	Short-time Fourier transform
